# A computational dynamic systems model for *in silico* prediction of neural tube closure defects^☆^^☆☆^

**DOI:** 10.1016/j.crtox.2024.100210

**Published:** 2024-12-18

**Authors:** Job H. Berkhout, James A. Glazier, Aldert H. Piersma, Julio M. Belmonte, Juliette Legler, Richard M. Spencer, Thomas B. Knudsen, Harm J. Heusinkveld

**Affiliations:** a Centre for Health Protection, National Institute for Public Health and the Environment, Bilthoven, the Netherlands; b Institute for Risk Assessment Sciences, Utrecht University, Utrecht, the Netherlands; c Biocomplexity Institute, Indiana University, Bloomington, USA; d Department of Physics, NC State University, Raleigh, NC, USA; e U.S. EPA/ORD, Research Triangle Park, NC, USA

**Keywords:** Neural tube closure, Multicellular agent-based model, Computer simulation, Developmental toxicity, *In silico*, Probabilistic assessment, Gene network

## Abstract

Neural tube closure is a critical morphogenetic event during early vertebrate development. This complex process is susceptible to perturbation by genetic errors and chemical disruption, which can induce severe neural tube defects (NTDs) such as spina bifida. We built a computational agent-based model (ABM) of neural tube development based on the known biology of morphogenetic signals and cellular biomechanics underlying neural fold elevation, bending and fusion. The computer model functionalizes cell signals and responses to render a dynamic representation of neural tube closure. Perturbations in the control network can then be introduced synthetically or from biological data to yield quantitative simulation and probabilistic prediction of NTDs by incidence and degree of defect. Translational applications of the model include mechanistic understanding of how singular or combinatorial alterations in gene-environmental interactions and animal-free assessment of developmental toxicity for an important human birth defect (spina bifida) and potentially other neurological problems linked to development of the brain and spinal cord.

## Highlights

• Developed a dynamic systems agent-based model of neural tube closure.
• Simulates mammalian neural tube closure using cell signaling and biomechanics.
• Predicts both the nature and probability of defects from genetic perturbations.
• Model predictions align with biological phenotypes observed in existing research.
• Promising tool for animal-free, probabilistic assessment of developmental toxicity.

## Graphical abstract

**Figure:**
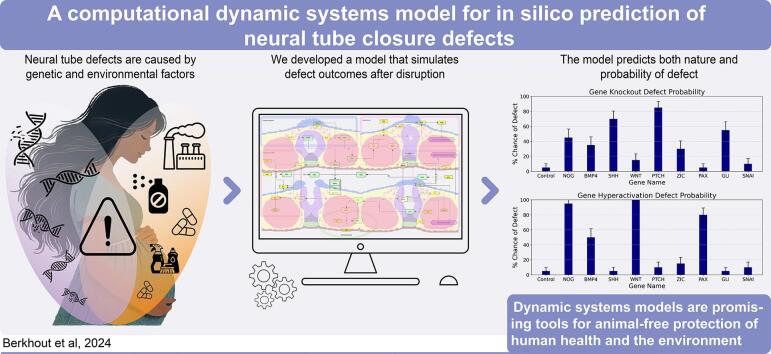

## Introduction

Critical to embryonic development of the brain and spinal cord is elevation and folding of the neural plate into a neural tube. This process, neural tube closure, occurs between day 23 and day 30 of human gestation (Gilbert et al.). Its failure underlies neural tube defects (NTDs) such as spina bifida and anencephaly. With an estimated global incidence of 20 cases per 10,000 live-births, NTDs are among the most prevalent human congenital malformations (Kancherla, 2023). Individuals born with NTDs are known to have severe neurological and developmental problems.

Neural tube closure is a tightly regulated process that involves the coordinated action of various signaling pathways and cellular processes. This system is susceptible to disruptions, including genetic variations that alter the normal function of key genes involved in the process, and environmental factors such as chemical exposures (Kancherla, 2023, Heusinkveld et al., 2021, Nikolopoulou et al., 2017). Exposure to chemicals, such as pharmaceuticals, pesticides, and industrial chemicals, increases the risk of NTDs (Felisbino et al., 2024, Brender et al., 2014, Yazdy et al., 2013, Rull et al., 2006, Mulu et al., 2022, Lupo et al., 2011, Isaković et al., 2022, Huang et al., 2023). Therefore, these chemicals warrant attention in safety assessments traditionally conducted in guideline prenatal development studies, such as OECD 414 and OPPTS 870.3700 (OECD, 2018, EPA, 1998). However, there is an increasing demand for alternative approaches due to ethical concerns, the call for greater chemical coverage with fewer resources, and scientific arguments related to the limited predictability of animal models for human health (Kiani et al., 2022, Piersma, 2006, Piersma et al., 2019).

Various *in vitro* alternatives for developmental toxicity testing have been developed during the past decades. These alternatives are often based on pluripotent stem cells, small model organisms such as the zebrafish, and complex organoid or *in vitro* engineered microsystems (Xue et al., 2018, Lee et al., 2010, Dreser et al., 2020, McCollum et al., 2011, Van Der Ven et al., 2022, Karzbrun et al., 2021, Xue et al., 2024, Knight et al., 2018). These approaches may replicate some key aspects of neural tube development (e.g., neuruloids, neural rosettes), offering a window into the potential teratogenic effects leading to NTDs. Despite advancements, it is still challenging to capture the complex interactions and integrative biology of *in vivo* mammalian development.

Computational agent-based models (ABMs) are a method in which individual entities, or agents—specifically cells in this context—are simulated to mimic and study complex behaviors. These models put cellular dynamics in motion to reproduce self-organizing and emergent phenotypes designed in a computer model to enable the prediction of systems-level outcomes linked to defined biomolecular abnormalities (Hutson et al., 2017, Kleinstreuer et al., 2013, Swat et al., 2012, Shirinifard et al., 2012). For example, the models developed for blood vessel formation and palatal fusion have demonstrated how genetic and chemical perturbations, represented by adjusted fold changes in key genes, can predict adverse outcomes observed *in vivo* (Hutson et al., 2017, Kleinstreuer et al., 2013). This approach provides valuable understanding of the mechanisms and vulnerabilities of these developmental processes. When designed with sufficient biological accuracy, ABMs can probabilistically assess the impact of perturbations on neural tube closure. This offers insights into mechanisms underlying neural tube closure and potential disruption of this process by chemical or genetic factors.

In this study, we present a new computational ABM that recapitulates the morphogenetic events driving mammalian neural tube closure, using the open source CompuCell3D modeling environment (Swat et al., 2012). Our ABM is based on the physiological map of mammalian neural tube closure we developed earlier, which systematically organizes the cellular and molecular interactions driving this process (Heusinkveld et al., 2021). This map serves as a reference tool for identifying critical pathways and integrating them into the ABM. Here, we show how variations in cell signals and responses may lead to alterations reminiscent of NTDs that can be calibrated to *in vivo* models (mouse, zebrafish). Additionally, the computer model can offer mechanistic insights often obscured in traditional animal models to bridge the gap between a molecular alteration and a developmental phenotype.

## Results

### A computational model of the mammalian neural tube closure

We built a multi-scale model of mammalian neural tube closure using CompuCell3D, an open-source platform that simulates multicellular systems based on a Cellular Potts model. The platform takes into account the specific behaviors of cells, their regulatory signals, and physical properties (Swat, et al., 2012). Neural tube closure involves complex tissue morphogenesis where tissue bending plays an essential role. This bending is primarily driven by apical constriction of cells in the neuroepithelium. The collective changes shape the tissue at emergent hinge points (bending points) (Nikolopoulou et al., 2017). The current model is two-dimensional in the dorso-ventral plane representing the mid-lumbar region of the neural tube. Within this region, both median hinge points (MHPs) and dorsolateral hinge points (DLHPs) are present during neural tube closure (Juriloff and Harris, 2018).

**Fig. 1:**
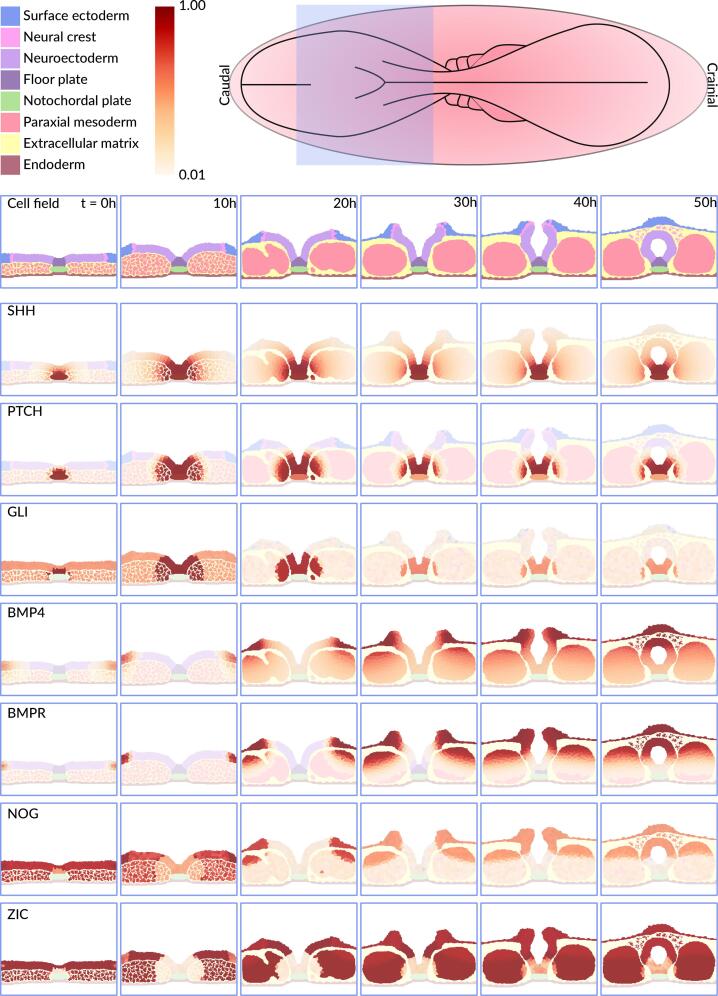

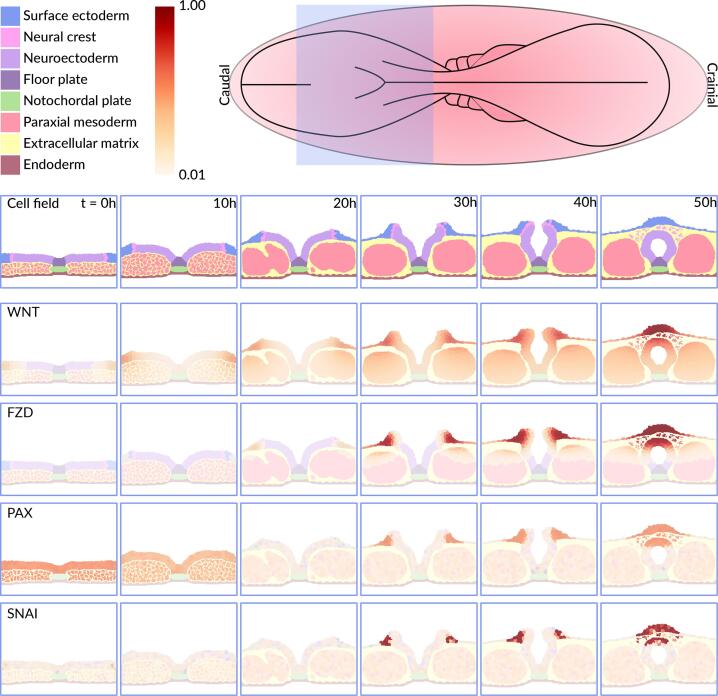
A computational model of mammalian neural tube closure. The 8 diﬀerent cell types featured in the model are incidated in legend with their respective color. The colorbar represents relative gene expression, where 1 indicates the highest amount and 0 the lowest amount. The model respresents a dorso-ventral section of the middle spinal region of the embryo, outlined by the violet box in the schematic. At t = 0 h the size of the model is 252 µm by 50 µm by 1. The gene that is visualized in each image series is indicated in the top left corner of the ﬁrst image of the series. (For interpretation of the references to color in this figure legend, the reader is referred to the web version of this article.)

**Fig. 2:**
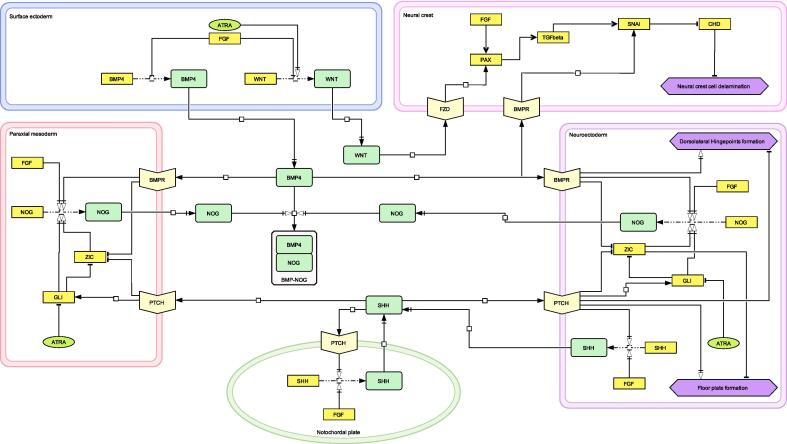
Gene regulatory network that drives the neural tube closure model. The network regulates three key processes crucial for successful neural tube closure (purple diamonds): floor plate formation, dorsolateral hinge point formation, and neural crest cell delamination. Symbols represent specific molecular and cellular components within the network: light-yellow chevrons indicate receptors, yellow rectangles represent genes, and green rectangles depict proteins. Arrows and their formatting denote specific interactions: white arrows represent specific triggering events; black arrows indicate receptor binding; and black arrows with a line signify transport processes. White squares provide anchor points for the attachment of trigger arrows to reaction pathways, enhancing diagram clarity. (For interpretation of the references to color in this figure legend, the reader is referred to the web version of this article.)

The stepwise progression of the model is shown in Fig. 1. It starts as a planar neural plate, measuring 252 µm by 50 µm by 1 µm, and over the course of 50 simulated hours undergoes the morphogenetic changes necessary to form a closed neural tube (Cork and Gasser, 2012). The start of the model approximates gestational day 28, Carnegie Stage 10 in human development. To make the model as human-like as possible, we adjusted its physical properties, time constraints and spatial organization to closely represent the characteristics of human neural tube closure (Cork and Gasser, 2012). Executing the computer model simulates key morphogenetic events akin to those seen in reconstructed sections of the embryo (Cork and Gasser, 2012). These events are directly driven by a gene regulatory network that is embedded in each cell featured in the ABM (Fig. 2). Below we describe the biological processes that occur during neural tube closure and are incorporated in our model, which progress through six distinct stages.

In the first stage, molecular protein signals shape the initial morphogenesis. The notochord starts to produce sonic hedgehog (SHH), which activates Patched Homolog 1 (PTCH1) (Aruga, 2004, Caspary and Anderson, 2003). PTCH1 downregulates Zinc finger proteins (ZIC) and upregulates Glioma-associated oncogene 2 (GLI2) proteins, which inhibit ZIC (Fig. 2) (Aruga, 2004, Caspary and Anderson, 2003, Copp and Greene, 2010). This starts apical constriction and the formation of median hinge-point cells in the targeted region (Caspary and Anderson, 2003, Copp and Greene, 2010). Opposing the SHH gradient is the Bone morphogenetic protein 4 (BMP4) gradient, secreted by the surface ectoderm (Copp and Greene, 2010, Liu and Niswander, 2005, Sheng et al., 2010, Anderson et al., 2016). BMP4 induces cell proliferation in the mesoderm and activates the BMP receptor (BMPR) (Sheng et al., 2010, Anderson et al., 2016, Chesnutt et al., 2004, Sakurai et al., 2012). BMP4 activity is regulated by fibroblast growth factor 8 (FGF8) and Noggin (NOG). FGF8, which levels are highest in the first stage, directly inhibits BMP4 gene expression (Fig. 2). NOG, secreted by the mesoderm, neuroectoderm, and notochord, acts as a ligand that binds to BMP4, forming an inactive NOG-BMP4 complex (Fig. 2) (Felder et al., 2002, Phan-Everson et al., 2021, Zimmerman et al., 1996). The surface ectoderm also secretes WNT, which activates the Frizzled (FZD) receptor to start the cascade of events leading to neural crest cell delamination (Nikolopoulou et al., 2017, Gray et al., 2013, Pera et al., 2014). However, early neural crest specification and delamination is inhibited by the high levels of FGF8 (Diez Del Corral and Morales, 2017). This is complemented by low levels of all-trans retinoic acid (ATRA), FGF8′s regulatory counterpart (Heusinkveld et al., 2021).

In the second stage, approximately 10 h into neural tube development, the median hinge point cells anchor to the notochord beneath them and start to form the floor plate. The floor plate gives stability to the neural plate and starts to produce SHH, reinforcing the sustained SHH gradient along the ventral-dorsal axis (Greene et al., 1998). Floor plate formation in combination with further growth of the mesoderm results in the formation of a furrow at the midline of the model, the neural groove (van Straaten et al., 1993). Meanwhile, physical forces outside of the neural plate are also at work. The surface ectoderm starts to push towards the midline of the neural tube (Schoenwolf and Alvarez, 1991). These forces are generated by the pressure of surrounding tissues or fluids and are critical for bending of the neural plate later in time. In our simulation, we approximated this behavior by applying a directional vector force on the surface ectoderm, which compels the surface ectoderm to move inward. This ensures that the neural tube invaginates into the embryo and not outward (Schoenwolf and Alvarez, 1991).

In the third stage, around 20 h into development, key molecular changes occur. ATRA levels continue to increase, exceeding FGF levels, while FGF levels further decrease (Heusinkveld et al., 2021). This shift results in reduced NOG levels, subsequently increasing the amount of free active BMP4. Still, the NOG-BMP4 balance is maintained because BMP4 induces the production of NOG (Felder et al., 2002, Phan-Everson et al., 2021). Somitogenesis commences and plays a critical role in shaping and stabilizing the neuroectodermal tissue. This contributes to the elevation of the neural folds by exerting physical forces that push up the neuroectoderm, thereby deepening the neural groove (Christodoulou and Skourides, 2023). During neural tube closure, somite formation is initiated via an oscillatory mechanism, often referred to as the “segmentation clock”, in which the Notch, WNT and FGF pathways play a major role (Gibb et al., 2010, Maroto et al., 2012). The computer model abstracts a segmentation clock without its regulators, as noted in the Methods section.

Around 30 h into development, the hallmark of the fourth stage is marked by the formation of dorsolateral hinge-points (DLHPs) at the neural plate's upper regions. DLHP formation initiates bending of the neural plate via apical constriction, the formation of wedge-shaped cells. This process is driven by localized actomyosin contraction at the apical side of cells and is accompanied by cell elongation, as cell volume is redistributed along the apicobasal axis (Sawyer et al., 2010). Basal thickening, with nuclei retained basally, further reinforces this wedge shape and stabilizes the hinge point structure. DLHP formation is highest in regions with intermediate BMP4 levels, where BMP signaling is inhibited by NOG, ensuring proper spatial localization (Gilbert et al.). In contrast, SHH signaling via PTCH acts as an inhibitor of DLHP formation, further refining their placement (Stottmann et al., 2006, Ybot-Gonzalez et al., 2007). At this stage, the continuous downregulation of FGF and increasing activity of ATRA allows the start of the neural crest cell delamination cascade. The end of the cascade results in an E- to N-cadherin switch, which allows neural crest cells to delaminate. In our model we simplified this through a single Cadherin (CAD). When CAD expression is high, its properties approximate E-cadherin. Low expression switches to N-cadherin properties. The E/N Cadherin switch is inhibited by FGF8 and induced by BMP4, FZD, paired box gene 3 (PAX3) and snail family transcriptional repressor 1 (SNAI1), which is increasing in this stage (Fig. 1) (Gray et al., 2013, Pera et al., 2014, Ichi et al., 2010, Nakazaki et al., 2009).

In the fifth stage, around 40 h into development, the formation of DLHPs further progressed into the center region of the neural plate under the influence of an increasing BMP4 gradient (Sheng et al., 2010, Anderson et al., 2016). This change, combined with the physical forces exerted by the surface ectoderm towards the midline, leads to near fusion of the surface ectoderm and neuroectoderm, almost closing the neural tube (Schoenwolf and Alvarez, 1991). The rising FZD, PAX3 and SNAI1 levels cause the start of the neural crest cell delamination (Gray et al., 2013, Pera et al., 2014, Ichi et al., 2010, Nakazaki et al., 2009).

After approximately 40–50 h of development, a section of the neural tube closes. The final stage displays 50 h into development, the neural tube is now fully closed, and the neural crest cells delaminated. A summary of all the signaling molecules and cell behaviors implemented in the computational model is presented in Table 1.

**Table 1:** Summary of the molecules and behavior in the neural tube closure model.

Cell type	Behavior	Signal molecules	Reference
Surface Ectoderm	BMP4 secretion, WNT secretion, cell migration	BMP4, FGF8, ATRA, WNT	(Liu and Niswander, 2005, Sheng et al., 2010, Anderson et al., 2016)
Neural crest	cell delamination	WNT, FZD, FGF8, PAX3, TGFbeta, SNAI1, CHD, BMP4, BMPR	(Gray et al., 2013, Pera et al., 2014, Ichi et al., 2010, Nakazaki et al., 2009)
Neuroectoderm	apical constriction, DLHP formation, floor plate formation, NOG secretionSHH secretion	NOG, FGF8, BMP4, BMPR, ZIC, GLI2, ATRA, SHH, PTCH	(Greene et al., 1998, van Straaten et al., 1993, Caspary and Anderson, 2003, Copp and Greene, 2010, Felder et al., 2002, Phan-Everson et al., 2021, Zimmerman et al., 1996)
Mesoderm	cell proliferation, somite formation,NOG secretion	NOG, FGF8, BMP4, BMPR, ZIC, GLI2, ATRA, SHH, PTCH	(Anderson et al., 2016, Chesnutt et al., 2004, Sakurai et al., 2012, Felder et al., 2002, Phan-Everson et al., 2021, Zimmerman et al., 1996)
Notochordal plate	SHH secretion, Floor plate induction	SHH, PTCH, FGF8	(Aruga, 2004, Caspary and Anderson, 2003)

### Towards modeling neural tube closure defects

**Fig. 3:**
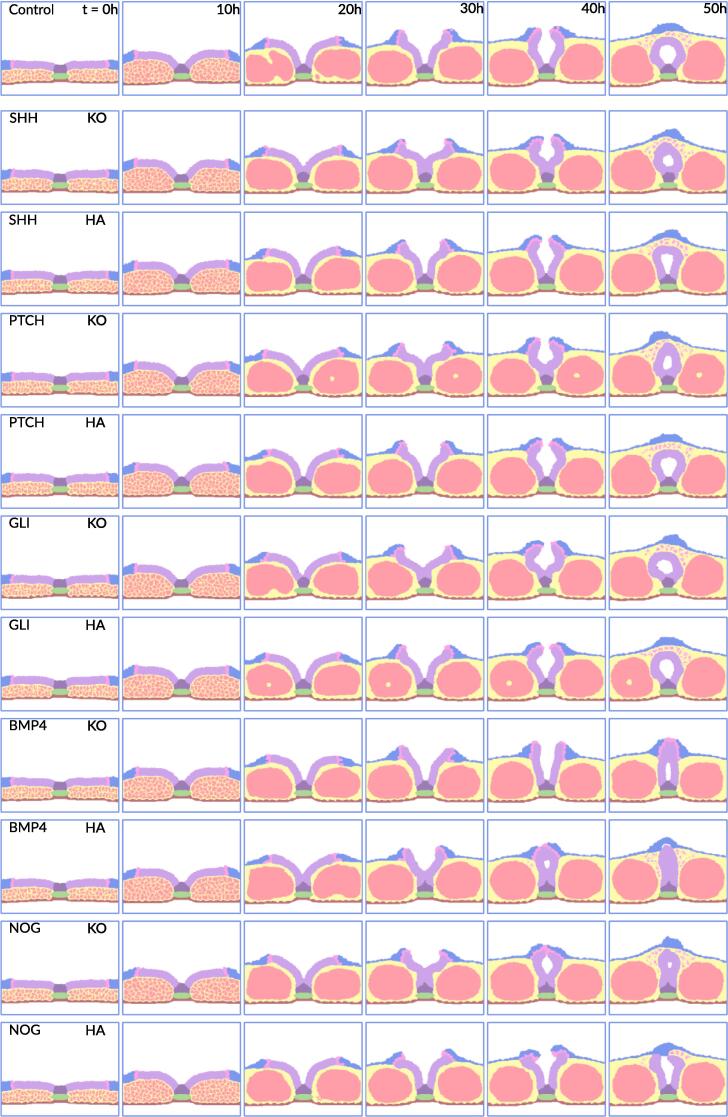

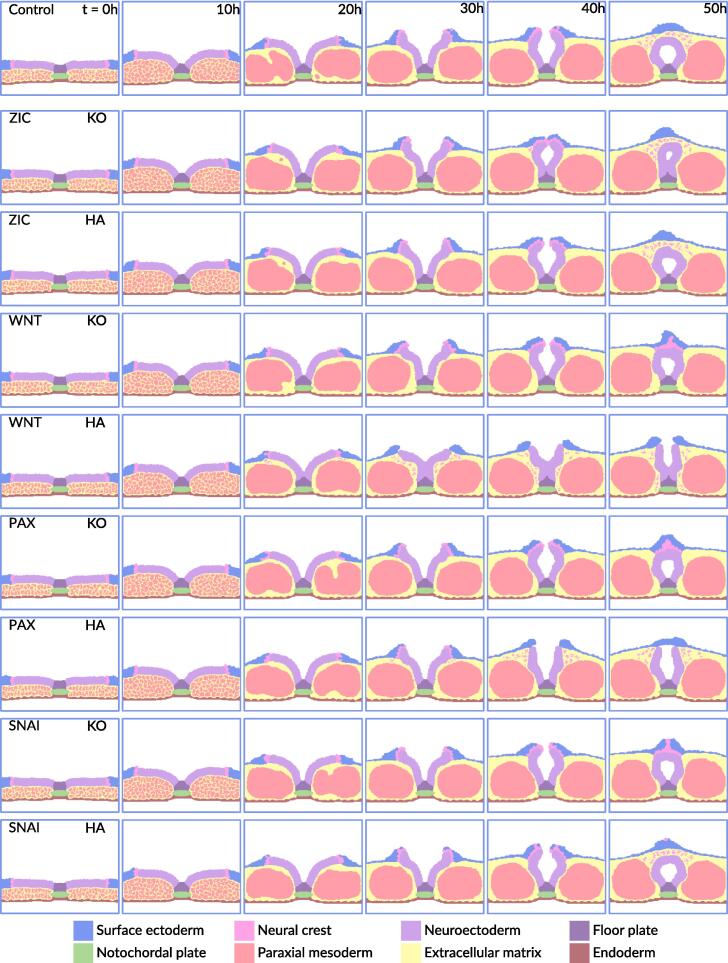
Towards modeling neural tube closure defects. The gene that is perturbed is noted in the top left corner of the ﬁrst image of the series. Gene knockout is indicated with KO and gene hyper- activation with HA, in the top right of the ﬁrst image in the series. The 8 diﬀerent cell types featured in the model are incidated in legend with their respective color. The extracellular matrix can be captured within the somite randomly, which is not a result of the perturbation scenario.

To calibrate our computational model against known biological data and explore potential developmental anomalies, we performed *in silico* parameter variation experiments. In these experiments nine different genes that participate in the regulation of the three key processes (floor plate formation, DLHPs formation, neural crest cell delamination) were hyperactivated or knocked out, while keeping the rest of the parameters the same. Each hyperactivation or knockout scenario was simulated 20 times to calculate the probability of defect, with the standard error of the mean (SEM) calculated to represent variability across simulations. The results presented in the following sections are organized per morphogenetic event and offer insight in the predicted dynamics of neural tube closure. The series of images shown are examples of the most common outcomes (Fig. 3). A summary of the *in silico* gene perturbation experiments is presented in Supplementary Table 1.

### Floor plate formation

The floor plate is a specialized structure of the neural tube and is formed in the second stage of the neural tube closure. Floor plate formation was disrupted in the model by knockout of *SHH*, *PTCH1* or *GLI2* (Fig. 3). This prevented the neuroectoderm from anchoring to notochord, which reduced the stability of the neural plate. As a result, structural malformations occurred at the midline of the neural tube.

**Fig. 4:**
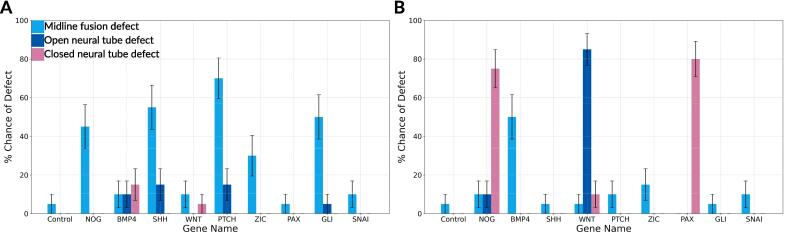
Incidence of identiﬁed neural tube closure defect types. (A) Binning of cumulative incidences into their respective defect types for all gene knockout scenario’s. (B) Binning of cumulative incidences into their respective defect types for all gene hyperactivation scenario’s. On the Y-axis the probability of a defect of the type to occur as predicted by the model. On the X-axis the gene that is perturbated. Error bars represent the standard error of the mean. Sky-blue: Midline fusion defect, cerulean-blue: Open neural tube defect, rosy-pink: Closed neural tube defect.

Due to disruptions in floor plate formation, the strength of the SHH gradient along the ventral-dorsal axis was reduced. Due to the reduced SHH gradient (the inhibitor of DLHP formation), DLHP formation was increased near the ventral side of the tube. Due to increased DLHPs formation, the neural tube still managed to close in most cases. However, the lack of floor plate and reduced stability of the neuroectoderm reduced the size of the neural canal (the hollow part of the neural tube). When the neural canal size was reduced by 50 % or more in our ABM, we considered a neural tube defect to be present. This threshold was selected as a conservative estimate to represent a substantial deviation from normal development, likely impairing growth and function of the spinal cord and associated structures. Such reductions increase the risk of conditions like congenital lumbar spinal stenosis, where a narrowed spinal canal present at birth can lead to severe neurological impairments (Ciricillo and Weinstein, 1993, Oskouian et al., 2007). While specific quantitative thresholds during embryogenesis are not well-established, clinical observations in adults link a neural canal size reduction of 30–50 % or more to lumbar spinal stenosis (Gopinathan, 2015, Hughes et al., 2015). Therefore, we used a 50 % reduction as a biologically plausible threshold for identifying neural tube defects in our ABM. Based on this threshold, the increased probability of a neural tube defect following knockout of *SHH*, *PTCH1* and *GLI2* was estimated- by the model to be 70 % ± 11, 85 % ± 8 and 55 % ± 11 respectively (Fig. 4).

Studies with *Ptch1* and *Shh* null mouse embryos report a disrupted floor plate formation with a spatial organization similar to the prediction of our model (Ybot-Gonzalez et al., 2002, Chiang et al., 1996, Murdoch and Copp, 2010). A lack of floor plate and increased DLHP formation indicates that these genes play a role in the inhibition of DLHP formation. Although the neural tube closure disruptions appear quite severe, the neural tube was able to close in most cases, both in our model as well as in other studies (Ybot-Gonzalez et al., 2002, Chiang et al., 1996). However, due to the severe spatial disruptions observed, especially at the midline, we do not consider the neural tube to be functional under the aforementioned knockout scenarios. In both humans and mice, mutations in *SHH* have been found to cause holoprosencephaly. This indicates that while the notochord extends only rostrally to the midbrain and the floor plate may not be essential for the initial closure of the neural tube, these features are critical for the subsequent stages of forebrain development (Chiang et al., 1996, Roessler et al., 1996, Rubenstein and Beachy, 1998). These holoprosencephaly features were also found in human loss-of-function GLI2 mutations, but less severe compared to *SHH* mutations, which is reflected in the predictions of our neural tube closure model (Roessler et al., 1996). Furthermore, GLI2 was found to be essential for floor plate formation in *Gli2* null mouse embryo’s (Matise et al., 1998). Mouse *Ptch1* null embryos die at approximately E9.5 likely due to a role of Ptch1 in heart development (Murdoch and Copp, 2010).

Our model simulations predicted an increased risk for closure defects of 5 % ± 5, 10 % ± 7, and 5 % ± 5 for *SHH*, *PTCH1*, and *GLI2* hyperactivation, respectively. However, the hyperactivation of these genes appeared to not cause a significant increased probability of NTDs (based on Chi-Square Test versus the control simulations). *SHH* hyperactivation resulted in a reduction of DLHP formation, but not sufficient to completely block it. This phenomenon has been found in mouse embryos that were exposed to additional Shh via an implanted Shh bead (Ybot-Gonzalez et al., 2002, Chiang et al., 1996). There was no literature that specifically investigated the effects of *PTCH1* hyperactivation on neural tube closure; however, given the relationship between SHH and PTCH1 we hypothesize that hyperactivation of *PTCH1* may have a comparable effect. This hypothesis aligns with the predictions of our model. Similarly, no studies were found that investigate the effects of *GLI2* hyperactivation on neural tube closure.

### Dorsolateral hinge point formation

DLHPs are critical regions in the neural tube where bending occurs to facilitate the closure process during the fourth stage of neural tube closure. DLHP formation was disrupted in the model by knockout of *BMP4*, *NOG* or *ZIC* (Fig. 3). Due to the absence of these bending points, the neural plate was not able to bend properly which resulted in an increased probability for neural tube defects to occur.

The increased significant NTD risk is 35 % ± 11, 45 % ± 11 and 30 % ± 10 for knockout of *BMP4*, *NOG* and *ZIC*, respectively (Based on Chi-Squared Test versus control simulations). DHLP formation is not aways essential for a neural tube closure. Studies have indicated that neural tube closure may shift to a different mode of closure (one in which DLHPs are not necessary) under certain perturbations scenarios in mice, a phenomenon also confirmed by our model (Juriloff and Harris, 2018, Ybot-Gonzalez et al., 2007).

Studies in *Bmp2*, *Bmp4* and *Nog* null mouse embryos report reduced DLHPs formation and a spatial organization similar to the predictions of our model (Stottmann et al., 2006, Ybot-Gonzalez et al., 2007). Loss of function mutations of these genes have been detected in human spina bifida patients, highlighting the importance of these genes in human development (Felder et al., 2002).

In mice, a loss-of-function mutation in the *Zic2* gene leads to NTDs in 22 % of the embryos (Elms et al., 2003). These defects arise because *Zic2* normally increases *Nog* expression, which is important for proper DLHP formation. Interestingly, our ABM prediction of 30 % ± 10 aligns well with the biological findings, suggesting that the model captures key aspects of the underlying mechanisms. The slight difference could reflect factors such as the combinatorial action of multiple ZIC genes, which may amplify effects in our ABM, or the specific nature of the mouse mutation, which involves only one Zic gene and may result in a less severe phenotype. Additionally, ZIC2 plays a role in the delamination of neural crest cells, a function not included in our model (Elms et al., 2003, Heusinkveld et al., 2021, Escuin et al., 2023).

Hyperactivating *BMP4*, *NOG*, or *ZIC* increased the chances of neural tube defects by 50 % ± 11, 95 % ± 5, and 15 % ± 8, respectively. In our model, hyperactivated *BMP4* resulted in a midline fusion of the neural folds. This phenotype may be due to reduced DLHPs and increased mesodermal growth, since the somites seem slightly larger compared to control simulations. This outcome is similar to that observed in the *NOG* knockout, where the absence of the *BMP4* antagonist leads to increased *BMP4* activity. Although direct BMP4 hyperactivation experiments are unavailable, the removal of an antagonist like *NOG* can produce effects similar to hyperactivation of *BMP4*, supporting our model predictions (Stottmann et al., 2006, Ybot-Gonzalez et al., 2007).

The spatial organization of our model closely aligns with that of mouse embryos with hyperactivated *Nog* induced via implanted beats (Stottmann et al., 2006, Ybot-Gonzalez et al., 2007). In these cases, active Bmps quickly becomes bound by excess Nog, leading to major disruptions in patterning. Moreover, this interaction slows mesoderm growth, which BMP normally promotes, thus widening the gap between the neural folds. NOG severely reduced DLHPs formation, except at the upper dorsal side of the neural tube.

Hyperactivation of *ZIC* resulted in a slight increase in probability for a NTD to occur. This perturbation caused DLHPs to develop slightly later in time (Fig. 3). However, this was in most cases not enough to result in an NTD. Unfortunately, there was not data available to validate our model predictions.

### Neural crest cell delamination

Neural crest cell delamination is the process by which these cells detach from the neural tube to migrate and differentiate into various cell types during embryonic development. Loss of function of *WNT*, *PAX3*, *SNAI1* or *BMP4* caused complete inhibition of neural crest cell delamination in the model. Under these perturbations, the neural tube still managed to close normally. Neural crest cell delamination abnormalities have been shown in mouse *Pax3* null abnormalities in line with our predictions (Palmer et al., 2021, Zhou and Conway, 2016). This suggests that *PAX3* is essential for proper neural crest cell delamination.

The computer model predicts *SNAI1* to be essential for neural crest cell delamination. This is in concordance with studies done in Chick embryo and Xenopus, but not with studies in mouse (Murray and Gridley). Currently there is no information available to confirm the need for *SNAI1* in human neural crest cell delamination.

Besides DLHPs formation, BMP4 also plays a role in neural crest cell delamination via the BMPR receptor. Activation of BMPR increases transcription of *SNAI1* in the model, which is predicted by the model to be essential for neural crest cell delamination. As expected, knockout of *BMP4* in the model caused inhibition of neural crest cell delamination. This has been reported in a study using chick embryos (Piacentino et al., 2021). As mentioned earlier, it is unknown whether this prediction reflects neural crest cell delamination in human, as *Snai1* was found to not be essential for neural crest cell delamination in mouse studies (Murray and Gridley).

WNT is critical for neural tube closure and later stages of embryo development (Nikolopoulou et al., 2017, Patapoutian and Reichardt, 2000). Disruption in the WNT/PCP pathway have been linked to NTDs in human (Wang et al., 2019). Given the absence of studies identifying disruptions in *WNT*, we hypothesize that its essential role in early development prevents embryos with a *WNT* loss of function from developing to the stage where neural tube closure occurs. However, due to WNT’s crucial role in neural crest cell delamination, our model predicts that disruption in WNT signaling, possibly caused by external factors, could interfere with this process. Such external factors includes exposure to certain chemicals, such as triazole fungicides, metals, and certain pharmaceuticals (Wang et al., 2023, Yonezawa et al., 2018, Stewart et al., 2004, Koval et al., 2014, Mook et al., 2017, Cerrizuela et al., 2020).

Hyperactivating *WNT*, *PAX3*, and *SNAI1* lead to a 100 % ± 0, 80 % ± 9, and 10 % ± 7 chance of NTDs to occur, respectively. In cases of *WNT* and *PAX3* hyperactivation, these NTDs stem from early delamination of neural crest cells, as shown in Fig. 3. Because neural crest cells and the surface ectoderm exert physical forces together, their early separation can reduce these forces, potentially increasing the likelihood of NTDs as predicted by our model. We were not able to directly validate the model prediction for *WNT* and *PAX3* due to lack of gene hyperactivation experiments. However, there is a strong correlation between neural crest abnormalities and NTDs (Palmer et al., 2021, Zhou and Conway, 2016, Piacentino et al., 2021). Hyperactivation of *SNAI1* only slightly raises the chance of NTDs, and we do not observe clear early neural crest cell delamination. We were not able to validate this prediction due to lack of experimental data.

### Modeling distinct types of neural tube closure defects

With our synthetic gene perturbation models, we were able to identify three different types of defects: (i) overt neural tube defect (Spina bifida Aperta), where the surface ectoderm is not closed and the neural tube is exposed to the amniotic fluid;(ii) covert neural tube defect (Spina bifida occulta) where the neural tube is not closed, but the surface ectoderm is; and (iii) midline fusion defect, where the neural folds are fused in the midline. The incidence of these different types is visualized in Fig. 4, where we binned the cumulative incidences into their respective defect type for all the perturbation scenarios.

In the gene knockout scenarios, we found midline fusion defects most often. These were most present in *NOG*, *SHH*, *PTCH1*, *ZIC* and *GLI2*. With BMP4 knockout the defect distribution is roughly equal. *PAX3* and *SNAI1* knockout did not have a significant increase in defect probability. In the gene hyperactivation scenarios, there was more variation in the most present defect types. The model predicted a closed neural tube defect in *NOG* and *PAX3* 75 % ± 10 and 80 % ± 9 respectively. In *WNT* the most present defect was an open neural tube defect. In *BMP4* hyperactivation the most present defect type was midline fusion defect. In *ZIC* hyperactivation a minor increase in midline fusion defects was found (15 % ± 8). *SHH*, *PTCH1*, *GLI2* and *SNAI1* hyperactivation did not show a significant increase in neural tube defect probability.

## Discussion

We built and tested an ABM of neural tube closure based on multiple complex cell behaviors such as apical constriction, tissue bending and fusion. These cell behaviors are driven by a gene regulatory network of which the protein expression is comparable to those observed in various *in situ* hybridization experiments (Aruga, 2004, Ybot-Gonzalez et al., 2007, Ybot-Gonzalez et al., 2002, Chang et al., 2008, Liu and Jessell, 1998, Hirsinger et al., 1997).The underlying gene regulatory network allowed us to simulate potential outcomes (or “what-if” scenarios) related to perturbations in caudal neural tube closure. These perturbations were simulated as changes in gene expression within the model. The model predicted defect types reminiscent of biological phenotypes identified in literature. The primary drivers of these defects were identified as disruptions in floor plate formation, disruptions in DLHPs formation and disruptions in neural crest cell delamination.

Our ABM was able to differentiate between three distinct types of defects: Spina bifida occulta, Spina bifida aperta and midline fusion defects, providing probability predictions for the likelihood of each defect occurring. By running multiple simulations of a given genetic perturbation scenario, our ABM estimates the probability of that perturbation to induce various neural tube defects. This probabilistic approach stems from the stochastic elements integrated into our model, particularly in cellular behaviors such as apical constriction, cell motility, and other cell–cell interactions. While the gene regulatory network itself is deterministic, using fixed parameters for gene interactions, these stochastic cellular processes introduce variability across simulations. This variability enables the model to capture the inherent randomness of biological systems, simulating diverse developmental outcomes under similar conditions. As such, this approach allows for a more comprehensive understanding of the variability and uncertainty in biological responses, offering a nuanced risk evaluation that is particularly valuable for regulatory and safety contexts. However, validating these probability-based predictions remains challenging due to limitations in available quantitative data. Despite this, the qualitative similarities between the spatial organizations observed in our simulations and those found in *in vivo* tissue sections demonstrate the model’s potential to capture critical morphogenetic events and defects related to neural tube closure.

While our model offers significant insights into the mechanism of neural tube closure, there are a number of constraints, including those arising from model simplifications. For example, genes that are members of the same gene family are combined into a single representative gene and certain regulatory genes present in the physiological map are still omitted from the gene regulatory network. Similarly, signaling pathways responsible for cytoskeletal dynamics, such as those governing cell adhesion and movement, were abstracted, and incorporated as hard-coded behaviors (e.g. directional vector forces, pseudo energy functions). These simplifications allowed us to focus on pathways that determine successful neural tube closure, such as those regulating floor plate formation, DLHP formation and neural crest cell delamination.

Besides this, our model is designed as a 2D framework, focusing on a dorsal–ventral axis slice. This design choice, aimed at simplicity and relevance, allows for the efficient simulation of critical aspects like neural fold elevation, bending, and fusion, without the computational demands of 3D simulations. In this 2D framework, the rostro-caudal gradients ATRA and FGF are constant across the model space but vary over time. While this approach effectively captures the necessary signaling patterns for the simulated morphogenetic events, it simplifies their spatial dynamics, which are more prominent in a 3D context.

Despite these simplifications, our model captures the key cell behaviors essential to neural tube closure and includes the main pathways necessary to predict defects arising from disruptions in related gene expression. While processes like somite formation, cell adhesion and cell movement remain simplified, they could be refined in future iterations to improve the comprehensiveness of our model. Future expansions could also integrate additional pathways, such as those regulating purse-string formation and of zippering the surface ectoderm to capture closure mechanisms more extensively. Transitioning to a 3D model could offer a more realistic representation of neural tube closure by methodically expanding the 2D structure into a multi-layered configuration. This advancement could enable exploration of complex processes like elongation, rostral-caudal patterning, and torsion, which are beyond the scope of the current 2D model.

Parameterizing biological models is inherently challenging due to often incomplete, unreliable, or unavailable quantitative data. Few half-maximal constants or Hill coefficients are known for specific gene interactions, and assuming values for unknown parameters can introduce unpredictability into the system. Instead of striving for precise parameterization, we focused on creating a stable and functional system capable of capturing the qualitative behavior of biological processes. By standardizing the parameters in our gene regulatory network (as detailed in the Methods section), we ensured consistency and computational efficiency in the model. This approach balances biological plausibility with computational tractability, allowing the model to effectively represent gene regulation without the need for precise parameter values.

Similarly, we normalized and scaled protein gradients between 0 and 1 by assigning identical diffusion and decay constants for all gradients. Although these parameters may not be quantitatively accurate, the emergent behavior of the model mimics *in situ* hybridization data of neural tube closure observed at critical time points (Ybot-Gonzalez et al., 2007, Ybot-Gonzalez et al., 2002, Hirsinger et al., 1997). This qualitative approach ensures that the model captures biologically relevant behaviors without becoming entangled in uncertainties surrounding individual parameter values.

While Boolean models are commonly used for their stability and simplicity, integrating a Boolean framework into an ABM at the cellular level introduces significant computational cost. This is because Boolean logic must be resolved for each cell and every gene interaction within the system. By using scaled Hill functions with standardized parameters, our approach circumvents this issue, preserving the qualitative behavior and stability associated with Boolean models without the prohibitive computational cost.

This pragmatic approach is well-suited for exploring the conceptual feasibility of building a complex, stable biological model. However, it highlights the need for future studies to conduct sensitivity analyses and refine parameter estimates as more quantitative data become available.

Other challenges stem from the limited availability of mechanistic data specific to the human neural tube. Hence, the current model predominantly relies on data derived from animal studies. Although neural tube closure is a highly conserved process across vertebrates, the use of animal data as a primary source introduces a layer of complexity and potential variability (Heusinkveld et al., 2021). This reliance on non-human data may lead to unforeseen irregularities in the model predictions as subtle species-specific differences in gene regulation or expression might not be fully captured. Although the conserved nature of this developmental process supports the relevance of data derived from animal models, the extrapolation to human biology should be handled with care. Therefore, any conclusions drawn from our model should consider the potential discrepancies and limitations imposed by the interspecies differences. The ongoing acquisition and integration of human-specific data into our model will be important for refining its accuracy and predictive power.

Although our model has primarily been tested with genetic perturbations, it also highlights the potential effects that chemical disturbances could have on neural tube closure. Chemical exposures that interfere with key morphogenetic events such as floor plate formation, DLHPs formation and neural crest cell delamination, could lead to the defects similar to those predicted by our model. For example, teratogenic compounds like imidacloprid, cyclopamine, cytochalasin D, triazole fungicides and lead are known to disrupt critical signaling pathways involved in these events, causing defects that resemble those observed in our model simulations of gene knockouts (Fig. 3) (Liu et al., 2016, Ribes et al., 2010, López-Escobar et al., 2018, Menegola et al., 2005, Da Costa et al., 2021). These similarities suggest that our model could be adapted to evaluate the risks posed by various chemicals, making it a valuable tool for toxicological risk assessment.

In future applications for toxicity testing, our ABM could use bioactivity profiles related to chemical exposures measured in dedicated *in vitro* systems to predict the probability of causing neural tube defects. Suitable *in vitro* systems include human pluripotent stem cells differentiated to neuroectoderm, neural crest or surface ectoderm cell types (Xue et al., 2018, Lee et al., 2010, Nakanoh et al., 2024). Within these systems, gene expression changes caused by chemical exposure can be detected using techniques like RNA sequencing (RNA-seq) and quantitative PCR (qPCR). The effect of these gene expression changes on neural tube closure can then be simulated in our model. This approach offers mechanistic insights that are often obscured in animal models, where directly measuring neural tube closure is difficult, and outcomes like pup resorption are used as indirect indicators. By predicting the probability of human-relevant health effects, our model enhances our ability to detect potential developmental toxicities that might otherwise go unnoticed in rodent studies. This represents a significant step towards reducing reliance on animal testing, aligning with ethical standards, and ultimately improving human health outcomes.

## Methods

### Modeling software details

The neural tube closure model was developed using CompuCell3D, version 4.3.2 with scripting in python v3.9.1 (Swat, et al., 2012). CompuCell3D is an open-source environment for simulating multicellular systems given specified cell behavior, regulatory signals and physical properties. It uses the Glazier-Graner-Hogeweg model (also known as the cellular Potts model) that treats individual cells as autonomous agents which interact in a shared physical environment, modeled as a lattice consisting of lattice sites (voxels), in which each cell occupies multiple lattice sites (Shirinifard et al., 2012, Glazier and Graner, 1993). In our model, one lattice site represents 1 µm, and the environment consists of a 252 × 200 lattice grid. These parameters were derived from observations in the Virtual Human Embryo project, ensuring that the ABM aligns with the biological scale while having sufficient space for simulated growth (Cork and Gasser, 2012). The model dynamics employ a Metropolis Monte Carlo algorithm, which continuously tries to alter the cell occupying randomly selected lattice sites. The success of these attempts depends on the changes in a pseudo energy function. Specifically, changes that improve the pseudo energy always succeed, while those that worsen it may succeed based on a probability linked to the effective “temperature” (Teff), which was set to 10 in our simulations. This value was chosen based on recommendations in the CompuCell3D manual to balance stochasticity and energy minimization. This temperature introduces randomness in cellular motility and behavior, ensuring the model captures biological variability while preserving realistic dynamics (Swat et al., 2012). Exact details of the modeling framework can be found in the Compucell3D manual on compucell3d.org and in Shirinfiard et al. 2012 (Shirinifard et al., 2012). We quantified the dynamics in terms of Monte Carlo Steps (MCS), with each MCS involving 252 * 200 = 50400 change attempts. Each simulation ran for a total of 12.000 Monte Carlo Steps (MCS), with one MCS representing 15 s real time for a total real time of 50 h. Up to 20 simulations were simultaneously ran locally on a 3.60 GHz intel core i7-12700KF processor, which took approximately 2 h to complete. We stored cell and field-lattice configurations every 25 simulated minutes (100 MCS) and rendered the snapshots using a home-written python script (Fig. 1, Fig. 3).

### Model scope

Our model recapitulates a single dorso-ventral section of caudal neural tube closure, specifically the middle spinal region at somite locations 15–20. The model covers major morphogenetic events that revolve around the all-*trans*-retinoic acid (ATRA)-related molecular pathways for neural tube closure and disruption (Heusinkveld et al., 2021). These events include apical constriction, Floor plate formation, DLHPs formation, neural fold elevation, neural crest cell delamination, surface ectoderm fusion and somite formation. Here Floor plate formation, DLHPs formation and neural crest cell delamination are actively regulated by a constructed gene regulatory network (Fig. 2). The model approximates a timeline from embryonic (E) days E8.5 − E10 in the mouse and gestational days 28–31, Carnegie Stage (CS) CS10 − CS12 in human development (Cork and Gasser, 2012). Initial model size of 252 x 50 was set based on CS10 sections of the caudal neural tube at the virtual human embryo project (Cork and Gasser, 2012).

## Model cell properties

We initialized the cell volume for the paraxial mesoderm, surface ectoderm, and neural crest cell types at 49, with a variance of plus or minus 10, and positioned them in a 7 by 7 µm layout. This setup mirrors observations from the Virtual Human Embryo project (Cork and Gasser, 2012). We calibrated the delta cell volume increase to reflect a 24-hour proliferation rate without inducing cell proliferation. For the neuroectoderm and median hinge point cell types, we divided each cell into basal, lateral, and apical sites, each spanning 7 by 7 µm. These cells, as observed in the Virtual Human Embryo images, seemed elongated, being roughly three times longer than other cell types. To keep the neural plate structure stable and intact during the simulation, a minimum cell width of 7 µm was essential, making our simulated neuroectoderm cells approximately 2 µm wider than their estimated biological size. We ensured the structural integrity of the neural plate by connecting apical and basal cells within the neuroectodermal layer with springs, simulating the role of tight junctions through the use of the Focal Point Plasticity plugin in CompuCell3D (Swat et al., 2012). We set the target distance between these points at 5 µm to create a stable neuroectodermal layer.

### Model cell behavior

**Apical Constriction in MHP formation:** Apical constriction in the MHP is directly induced by SHH signaling secreted by the notochordal plate. When neuroectodermal cells are in contact with the notochord, the high SHH signaling triggers apical constriction via PTCH (Fig. 2). When a cell undergoes apical constriction, the target distance of the mechanical springs on the apical side of the cell reduces from 5 µm to 0 µm, implemented in steps of 0.005 µm per MCS for stable dynamic transition. This reduction represents the constriction of the actomyosin machinery on the apical surface of the cell (Cork and Gasser, 2012, Greene et al., 1998, Sawyer et al., 2010). To simulate the elongation of cells during apical constriction, additional mechanical springs are introduced between the apical and basal sides of the cell. These springs extend the target distance of the basal compartment to 15 µm from an initial 9 µm, reflecting the elongation behavior during apical constriction (Cork and Gasser, 2012, Sawyer et al., 2010). Simultaneously, cell volume is dynamically redistributed among three compartments (apical, lateral, and basal), increasing the basal compartment's volume by 57 % while proportionally decreasing the apical compartment’s volume. This adjustment mimics basal thickening and the formation of wedge-shaped cells while maintaining the overall cell volume (Gilbert et al., Cork and Gasser, 2012).

**Apical Constriction during DLPHs formation:** Apical constriction during DLHPs formation in the neuroectoderm is an emergent feature induced by intermediate BMP4 levels and inhibited by SHH (Fig. 2). While the behavior of cells undergoing apical constriction during DLHP formation resembles that during MHP formation, the triggering mechanisms differ. the first model calculates the maximum probability of apical constriction (*max_ac_*) for each neuroectodermal cell at every MCS based on local BMP4 concentration using the equation:

(1) $$\max_{\mathrm{ac}}=4\left(\frac{C_{\mathrm{bmp}}^{8}}{0.5^{8}+{\mathrm{xC}}_{\mathrm{bmp}}^{8}}*\frac{0.5^{8}}{0.5^{8}+C_{\mathrm{bmp}}^{8}}\right)$$

Here *max_ac_* is the maximum apical constriction probability and *C_bmp_* is the local BMP4 concentration. Next the inhibition of apical constriction by SHH (shh_aci_) is calculated using the equation:

(2) $${\mathrm{shh}}_{\mathrm{aci}}=\frac{0.5^{8}}{0.5^{8}+C_{\mathrm{shh}}^{8}}$$

where *shh_aci_* represents represents the level of apical constriction inhibition by SHH and C*_shh_* is the local SHH concentration. The combined probability of apical constriction (*rate_ac_*) is then determined by multiplying the BMP4 and SHH effects:

(3) $$\mathrm{rat}e_{\mathrm{ac}}=max_{\mathrm{ac}}*{shh}_{aci}$$

This value (*rate_ac_*) is subsequently corrected for time using a Poisson distribution:

(4) $$p_{\mathrm{ac}}=1-{exp}^{-\left(\frac{\mathrm{rat}e_{\mathrm{ac}}}{4}\right)}$$

Here *p_ac_* represents the time-corrected probability of apical constriction. Upon successful apical constriction, the cell transitions one step closer to adopting a wedge-shaped morphology. If unsuccessful, the cell reverts one step towards its original shape. This stochastic approach captures the biological variability and dynamic nature of DLHP formation.

**The segmentation clock:** In our model, we implemented the segmentation clock via an internal “counter,” that triggers the start of somite formation after 17 h simulated time (Cork and Gasser, 2012). Cells from the paraxial mesoderm type have a 1/2500 chance to differentiate to a somite cell with different properties. This probability is increased by 1 %, each time a paraxial mesoderm cell contacts a somite cell. These parameters were arbitrarily chosen to ensure rapid somite formation while minimizing the amount of extracellular matrix (ECM) captured within these somites (Martins et al., 2009). This implementation allowed us to reduce the complexity of the model while maintaining the critical function of the somites in neural tube closure.

### A gene regulatory network that drives the neural tube closure model

To simulate the complex interplay between various genes and proteins during neural tube closure in our model, we created a gene regulatory network of neural tube closure that contains key genes. All genes and proteins implemented in the model are displayed in the gene regulatory network (Fig. 2). The gene regulatory network implemented in our model is a refined extraction from a larger network and embedded in each cell in the ABM (Heusinkveld et al., 2021). Our network regulates three key processes for successful neural tube closure: (Fig. 2) floor plate formation, DLHP formation and neural crest cell delamination (Fig. 2). The development and simulation of this gene regulatory network, was conducted entirely within the CompuCell3D environment. As mechanistic data of the human neural tube is scarce the network primarily relies on animal data.

(5) $${\mathrm{gene}}_{a}=\frac{x^{n}}{K^{n}+x^{n}}$$

(6) $${\mathrm{gene}}_{i}=1-\frac{K^{n}}{K^{n}+x^{n}}$$

In these equations:

• x represents the gene expression level of the regulating gene (activator or inhibitor).
• *K* is the half-maximal constant.
• *n* is the Hill coefficient.

The Hill functions in the network are scaled between 0 and 1, with *n* = 8 and *K* = 0.5. These parameter values were selected due to the absence of precise values in existing data. To incorporate the inherent variability and unpredictability of biological systems, a random background transcription level is included for each gene in the network.

Protein gradients that are known to be critical for neural tube include BMP secreted by the surface ectoderm, SHH from the notochord and floor plate, NOG from the mesoderm, neuroectoderm, and floor plate, and WNT from the surface ectoderm. The protein concentration of these gradients is scaled between 0 and 1, a similar scaling as the gene regulatory network. We normalized the protein gradients by selecting identical values for each gradient for the diffusion and decay constant. The diffusion constant for all protein gradients was parameterized to be 0.24 µm (Kancherla, 2023)/s. The decay constant was parameterized to be 3.33*10^-5^s^−1^ in all compartments but increased to 2.333*10^-4^s^−1^ in the medium compartment (all values scaled to the simulation’s time and space units). This ensured consistent behavior of these signaling molecules across different cell types and spatial locations. Then, we calibrated the secretion constants based on cell number to accurately reflect the biological context (exact parameter values can be found in the simulation’s parameter file). The rostro-caudal gradients: ATRA and FGF are fixed in space, meaning their concentrations are the same throughout the model, but vary over time. Specifically, FGF concentration decreases over time and ATRA increases, both scaled between 0 and 1. This design mimics the rostro-caudal protein balance of FGF-ATRA crucial for neural tube closure, and ensures that these gradients capture the temporal dynamics needed for patterning in this 2D framework (Heusinkveld et al., 2021).

## Disclaimer

The views expressed in this article are those of the authors and do not necessarily reflect the views or policies of the U.S. Environmental Protection Agency. Mention of trade names or commercial products does not constitute endorsement or recommendation for use. The review and editorial management of this manuscript were handled entirely outside the domain of the three co-authors who are members of the CRTOX Editorial Board (Piersma), the Associate Editor (Heusinkveld), and the Editor-in-Chief (Knudsen).

## CRediT authorship contribution statement

**Job H. Berkhout:** Conceptualization, Methodology, Software, Validation, Formal analysis, Investigation, Writing – original draft, Writing – review & editing, Visualization. **James A. Glazier:** Conceptualization, Methodology, Software, Validation, Supervision. **Aldert H. Piersma:** Conceptualization, Project administration, Funding acquisition, Writing – review & editing. **Julio M. Belmonte:** Methodology, Software. **Juliette Legler:** Project administration, Funding acquisition, Writing – review & editing. **Richard M. Spencer:** Conceptualization, Methodology, Software. **Thomas B. Knudsen:** Conceptualization, Methodology, Validation, Supervision, Writing – review & editing. **Harm J. Heusinkveld:** Conceptualization, Validation, Supervision, Writing – review & editing, Project administration, Funding acquisition.

## Declaration of competing interest

The authors declare that they have no known competing financial interests or personal relationships that could have appeared to influence the work reported in this paper.

## Data Availability

All data generated during this study is available in the BioStudies database (http://www.ebi.ac.uk/biostudies) under accession number S-ONTX34.
